# Corticosteroid injection for plantar heel pain: a systematic review and meta-analysis

**DOI:** 10.1186/s12891-019-2749-z

**Published:** 2019-08-17

**Authors:** Glen A. Whittaker, Shannon E. Munteanu, Hylton B. Menz, Daniel R. Bonanno, James M. Gerrard, Karl B. Landorf

**Affiliations:** 1 Discipline of Podiatry, School of Allied Health, Human Services and Sport, La Trobe University, Melbourne, Victoria 3086 Australia; 2 La Trobe Sport and Exercise Medicine Research Centre, School of Allied Health, Human Services and Sport, La Trobe University, Melbourne, Victoria 3086 Australia

**Keywords:** Corticosteroid injection, Plantar heel pain, Plantar fasciitis, Meta-analysis

## Abstract

**Background:**

Corticosteroid injection is frequently used for plantar heel pain (plantar fasciitis), although there is limited high-quality evidence to support this treatment. Therefore, this study reviewed randomised trials to estimate the effectiveness of corticosteroid injection for plantar heel pain.

**Methods:**

A systematic review and meta-analysis of randomised trials that compared corticosteroid injection to any comparator. Primary outcomes were pain and function, categorised as short (0 to 6 weeks), medium (7 to 12 weeks) or longer term (13 to 52 weeks).

**Results:**

A total of 47 trials (2989 participants) were included. For reducing pain in the short term, corticosteroid injection was more effective than autologous blood injection (SMD -0.56; 95% CI, − 0.86 to − 0.26) and foot orthoses (SMD -0.91; 95% CI, − 1.69 to − 0.13). There were no significant findings in the medium term. In the longer term, corticosteroid injection was less effective than dry needling (SMD 1.45; 95% CI, 0.70 to 2.19) and platelet-rich plasma injection (SMD 0.61; 95% CI, 0.16 to 1.06). Notably, corticosteroid injection was found to have similar effectiveness to placebo injection for reducing pain in the short (SMD -0.98; 95% CI, − 2.06, 0.11) and medium terms (SMD -0.86; 95% CI, − 1.90 to 0.19). For improving function, corticosteroid injection was more effective than physical therapy in the short term (SMD -0.69; 95% CI, − 1.31 to − 0.07). When trials considered to have high risk of bias were excluded, there were no significant findings.

**Conclusions:**

Based on the findings of this review, corticosteroid injection is more effective than some comparators for the reduction of pain and the improvement of function in people with plantar heel pain. However, corticosteroid injection is *not* more effective than placebo injection for reducing pain or improving function. Further trials that are of low risk of bias will strengthen this evidence.

**Registration:**

PROSPERO registration number CRD42016053216.

**Electronic supplementary material:**

The online version of this article (10.1186/s12891-019-2749-z) contains supplementary material, which is available to authorized users.

## Background

Plantar heel pain [1] is a common foot condition that occurs in adults, with prevalence estimates between 4 and 7% [2, 3]. Several interventions are used to treat plantar heel pain, although there is limited evidence to suggest which interventions are more effective [4]. Corticosteroid injection is often used to treat plantar heel pain [5] but there is limited high-quality evidence to support its frequent use.

Previous systematic reviews [6–10] have summarised the effectiveness of corticosteroid injection for plantar heel pain but they have limitations, such as; not incorporating meta-analysis [6, 9], only including studies that compared corticosteroid injection to specific comparators [7, 8, 10], and not evaluating the strength of the evidence using the Grading of Recommendations, Assessment, Development and Evaluation (GRADE) approach [6, 7, 10]. In addition, a Cochrane Collaboration review [11] that evaluated corticosteroid injection for plantar heel pain also has limitations. For example, the authors pooled data from the same intervention to different categories (e.g. for one trial, the comparator was categorised both as a control and an orthosis), reported pooled data from different outcome measures using mean differences (not standardised mean differences), and used fixed-effect models when random-effects models would have been more appropriate [12]. When previous reviews are considered together, the limitations outlined above reduce the validity of their findings.

Because corticosteroid injection is frequently used to treat plantar heel pain, it is important to provide healthcare professionals with a robust summary of the findings of randomised trials, including the strength of the evidence from these trials. Accordingly, the objectives of this review were to: (i) conduct a comprehensive review of the effectiveness of corticosteroid injection on pain (including ‘first step’ pain), function, and plantar fascia thickness; (ii) summarise the available evidence and provide pooled effect sizes with meta-analyses; and (iii) use GRADE to evaluate the strength of the evidence.

## Methods

This review conforms to the Preferred Reporting Items for Systematic Reviews and Meta-analyses (PRISMA) guidelines [13], and was prospectively registered on PROSPERO (ID = CRD42016053216).

### Selection criteria

Included studies had to be randomised trials (quasi-randomised trials were excluded) published in a peer-reviewed journal. Trials were included if they compared corticosteroid injection for plantar heel pain against any comparator (placebo or active treatment) and included at least one outcome measure for either pain (including ‘first step’ pain) or function. Trials were excluded if they compared two different corticosteroid injection techniques or provided co-interventions that were not provided to all groups.

### Search strategy

Electronic databases MEDLINE, CINAHL, SPORTDiscus, Embase and the Cochrane Library were searched for randomised trials published in any language. The search was originally conducted on December 1, 2016 and was updated on April 17, 2019 (Additional file 1). Complementary searches were conducted on Google Scholar and trial registries (e.g. http://clinicaltrials.gov/). Citation tracking was performed for identified trials and reference lists were scanned for trials that may have been missed in the original search.

### Data collection

Search results were exported into Endnote X7.2.1 (Thomson Reuters, New York, USA) and duplicates removed. Titles and abstracts of studies were independently screened by two authors (GAW and JMG), and studies that did not meet the inclusion criteria were excluded. Full-text articles were obtained for remaining studies and these were examined for eligibility based on the inclusion criteria.

A data extraction form was used to extract trial characteristics and outcome data. Primary outcomes were pain (including ‘first step’ pain) and function. One secondary outcome was included, which was plantar fascia thickness. Other information including variables affecting bias, adverse effects and characteristics of the corticosteroid injections were also extracted. One author (GAW) extracted data and a random sample of 25% of the trials were analysed by a second author (JMG) to ensure extracted data were error free. The mean, sample size and standard deviation of outcome measures at time-points categorised as *short term* (0 to 6 weeks), *medium term* (7 to 12 weeks) and *longer term* (13 to 52 weeks) were extracted. Attempts were made to obtain missing data by contacting authors. If no response was received, missing standard deviations were calculated based on *P* values if possible [14]. Any remaining trials for which standard deviations were not available were imputed using pooled standard deviations from other trials in the meta-analysis [15].

### Data handling and analysis

All data were synthesised and analysed using RevMan (Version 5.3. Copenhagen: The Nordic Cochrane Centre, The Cochrane Collaboration, 2014). Trials were grouped for meta-analysis based on the comparator intervention. For trials that used multiple measures to evaluate the same outcome (such as pain measured on separate questionnaires), the primary outcome measure was used. If more than two trials compared corticosteroid injection to the same comparator with the same time-points for outcome assessment, the data were pooled for a meta-analysis.

Due to the design variability of the included trials, an inverse-variance random-effects model was applied to all meta-analyses [12]. Outcome measures for which a higher score indicated less pain or improved function were multiplied by − 1 to provide common directionality of results. The relative treatment effect for each study was estimated by calculating the standardised mean difference (SMD), even if trials used the same outcome measure, to consistently present findings across different meta-analyses. The SMD was interpreted as having a small effect if approximately 0.2, a moderate effect if 0.5, a large effect if 0.8 and a very large effect if 1.3 [16]. Heterogeneity was investigated using the *Chi*^*2*^ and *I*^*2*^ statistics [17].

### Assessment of study quality

Risk of bias assessment was performed independently by two authors (SEM and DRB) using the Cochrane Collaboration tool for assessing risk of bias and disagreements were resolved by consensus meeting [14]. A trial was considered to have a high risk of bias if at least one of the criteria was rated high risk. To be considered low risk of bias, all criteria had to be rated low risk. Any trials not meeting these criteria were considered unclear. The agreement between reviewers was evaluated by calculating a weighted kappa coefficient [18] using the *kap* command in Stata (version 16.0, StataCorp LLC, College Station, TX). A sensitivity analysis was conducted that excluded trials considered to be at high risk of bias to assess the impact on the original meta-analysis.

Assessment of trial quality at the outcome level was undertaken using GRADE [19]. The criteria used to make judgements for each criterion are outlined in Additional file 2.

## Results

**Fig. 1:**
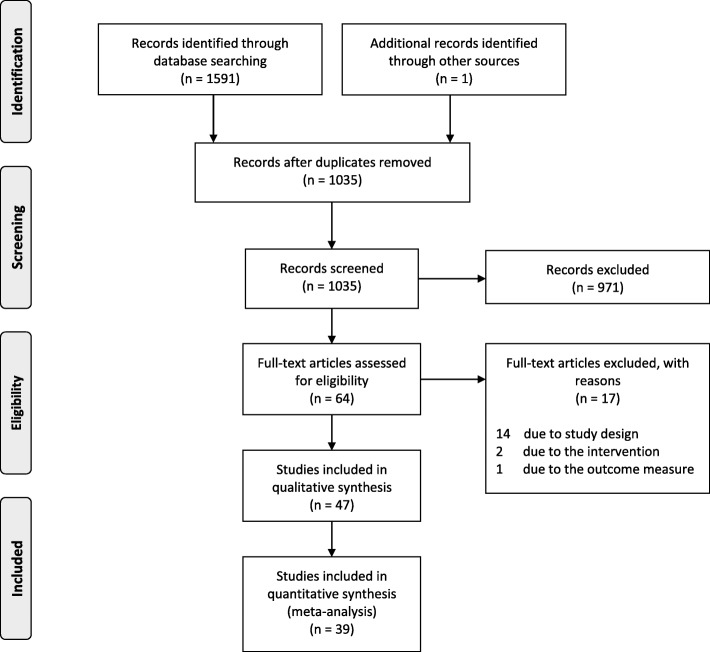
Flow of studies through the review

The systematic search identified 47 articles, and at the conclusion of screening, 47 individual trials were included in the final review (Fig. 1) [20–66]. Data were unable to be obtained from three trials [32, 48, 55] after contacting the authors, and five trials [33, 34, 37, 47, 53] could not be included in meta-analyses as the data were from composite outcome measures. Data from a four group trial [56] that sub-divided participants on the presence of perifascial oedema were combined to two groups so the data were similar to other trials. Finally, one trial [33] reported medians and interquartile ranges, which were converted to means and standard deviations [67].

The combined sample size from the included trials was 2989; 65.1% of participants were female, mean age 46.5 years and mean body mass index (BMI) 28.9 kg/m^2^. Each trial’s intervention, comparator, and participant characteristics are summarised in Table 1. The mean group size from the included trials was 28. Characteristics of the corticosteroid injections are summarised in Table 2; there were eight different types of corticosteroid used, with methylprednisolone acetate the most common (23/47 trials). Most trials (38/47) reported that they mixed a corticosteroid with a local anaesthetic and lidocaine was the most common (25/47 trials). A variety of injection techniques were used, most commonly without ultrasound guidance (35/47 trials) and by injecting at the point of maximal tenderness (14/47 trials).

**Table 1:** Descriptive characteristics of trials included in the review

Trial	Intervention	Comparator	Cointerventions	Participants per group		Female participants (%)	Mean age (years)	Mean BMI (kg/m^2^)	Duration of symptoms (weeks)^a^	Trial duration (weeks)	Trial setting
				Intervention	Comparator						
Abdihakin (2012) [20]	Corticosteroid injection	Placebo injection	i) Oral anti-inflammatory drugs three times daily; ii) stretches; iii) foot orthoses; iv) heel splints; v) shoe recommendations	44	38	52	42.9	31.7	NR	12	Outpatient clinic
Acosta-Olivo (2017) [21]	Corticosteroid injection	Platelet-rich plasma injection	Plantar fascia stretches	14	14	80	44.8	NR	> 12 weeks	16	Outpatient clinic
Afsar (2015) [22]	Corticosteroid injection	Autologous blood injection	None	62	61	57	31.8	NR	> 12 weeks	24	Outpatient clinic
Babaei-Ghazani (2019) [23]	Corticosteroid injection	Ozone injection	Plantar fascia and calf stretches	15	15	90	46.3	29.0	> 8 weeks	12	Outpatient clinic
Ball (2012) [24]	Corticosteroid injection	Placebo injection	Permitted to use analgesia if required	22	19	56	49.4	31.6	> 8 weeks	12	Rheumatology service
Celik (2015) [25]	Corticosteroid injection	Physical therapy	None	21	22	65	45.5	30.0	NR	52	Hospital
Crawford (1999) [26]	Corticosteroid injection	Local anaesthetic injection	NR	27	27	65	57.0	NR	NR	26	Hospital
	Corticosteroid injection + tibial nerve block	Local anaesthetic injection + tibial nerve block		26	26						
Diaz-Llopis (2012) [27]	Corticosteroid injection	Botulinum toxin-A injection	Plantar fascia and calf stretches	28	28	66	53.9	NR	> 26 weeks	4	Hospital
Elizondo-Rodriguez (2013) [28]	Corticosteroid injection	Botulinum toxin-A injection	Plantar fascia stretches	17	17	55	43.0	NR	> 12 weeks	26	Hospital
Eslamian (2016) [29]	Corticosteroid injection	Extracorporeal shockwave therapy	i) Foot orthoses and heel pads; ii) plantar fascia and calf stretches	20	20	82	42.1	NR	> 8 weeks	8	Hospital
Guevara Serna (2017) [30]	Corticosteroid injection	Extracorporeal shockwave therapy	NR	24	36	67	51.0	NR	> 12 weeks	52	Hospital
Guner (2013) [31]	Corticosteroid injection	Tenoxicam injection	A stretching and strengthening program	30	31	77	41.4	29.5	> 12 weeks	52	NR
Hanselman (2015) [32]	Corticosteroid injection	Cryopreserved human amniotic membrane	Plantar fascia and calf stretches	14	9	70	51.0	NR	> 12 weeks	18	NR
Hocaoglu (2017) [33]	Corticosteroid injection	Extracorporeal shockwave therapy	NR	36	36	87	49.0	28.7	> 26 weeks	26	Outpatient clinic
Hou (2018) [34]	Corticosteroid injection	Extracorporeal shockwave therapy	NR	39	38	35	41.5	25.4	> 12 weeks	26	Hospital
Jain (2015) [35]	Corticosteroid injection	Platelet-rich plasma injection	i) Eccentric stretches; ii) foot orthoses	22	24	65	55.6	NR	> 52 weeks	52	Hospital
Jain (2018) [36]	Corticosteroid injection	Platelet-rich plasma injection	Plantar fascia and calf stretches	40	40	42	38.3	24.1	> 12 weeks	26	Hospital
Johannsen (2019) [37]	Corticosteroid injection	Physical therapy	NR	31	30	58	45.0	26.2	> 12 weeks	104	University
		Corticosteroid injection + physical therapy			29						
Karimzadeh (2017) [38]	Corticosteroid injection	Control group	Plantar fascia stretches	12	12	67	47.5	NR	> 8 weeks	12	NR
		Autologous blood injection			12						
Kiter (2006) [39]	Corticosteroid injection	Autologous blood injection	NR	14	15	69	50.7	NR	> 26 weeks	26	University
Kriss (2003) [40]	Corticosteroid injection	Foot orthoses	NR	22	26	60	59.3	NR	NR	26	NR
		Corticosteroid injection + foot orthoses			31						
Lai (2018) [41]	Corticosteroid injection	Extracorporeal shockwave therapy	Acetaminophen as required	50	47	56	53.5	NR	> 4 weeks	12	Hospital
Lee (2007) [42]	Corticosteroid injection	Autologous blood injection	Plantar fascia and calf stretches	31	30	93	48.7	26.1	> 6 weeks	26	Outpatient clinic
Li (2014) [43]	Corticosteroid injection	Miniscalpel needle	Participants were permitted to continue with any conservative treatment	30	31	72	55.8	NR	> 26 weeks	52	Hospital
Mahindra (2016) [44]	Corticosteroid injection	Placebo injection	Plantar fascia and calf stretches	25	25	58	33.4	NR	> 12 weeks	12	NR
		Platelet-rich plasma injection			25						
Mardani-Kivi (2015) [45]	Corticosteroid injection	Extracorporeal shockwave therapy	None	41	40	84	44.3	29.6	< 6 weeks	12	University
McMillan (2012) [46]	Corticosteroid injection	Placebo injection	Plantar fascia stretches	41	41	48	52.6	31.1	> 8 weeks	12	University
Monto (2014) [47]	Corticosteroid injection	Platelet-rich plasma injection	i) CAM walker for two weeks; ii) Swedish heel drop program; iii) plantar fascia and calf stretches	20	20	57	55.0	29.2	> 16 weeks	104	NR
Mulherin (2009) [48]	Corticosteroid injection	Tibial nerve block		14	12	60	55 (median)	NR	NR	26	Community medical centre
		Corticosteroid injection + tibial nerve block			19						
Omar (2012) [49]	Corticosteroid injection	Platelet-rich plasma injection	NR	15	15	100	43.5	NR	NR	6	Hospital
Porter (2005) [50]	Corticosteroid injection	Extracorporeal shockwave therapy	Plantar fascia and calf stretches	64	61	66	39.2	NR	> 6 weeks	52	Hospital
Rastegar (2018) [51]	Corticosteroid injection	Dry-needling	NR	34	32	58	40.9	NR	> 12 weeks	52	University
Ryan (2014) [52]	Corticosteroid injection	Physical therapy	Calf stretches	28	28	57	49.3	25.2	> 52 weeks	12	University
Saber (2012) [53]	Corticosteroid injection	Extracorporeal shockwave therapy	NR	30	30	55	34.2	29.0	> 26 weeks	12	Outpatient clinic
Serbest (2013) [54]	Corticosteroid injection	Extracorporeal shockwave therapy	NR	15	15	53	45.2	30.5	> 6 weeks	12	Sports medicine clinic
Shetty (2019) [55]	Corticosteroid injection	Placebo injection	i) Oral enterocoxib and paracetamol for 5 days; ii) plantar fascia stretches; iii) eccentric calf strengthening	30	30	54	44.6	NR	> 12 weeks	78	Hospital
		Platelet-rich plasma injection			30						
Sorrentino (2008) [56]	Corticosteroid injection in participants with perifascial oedema	Extracorporeal shockwave therapy	NR	16	15	56	NR	27.9	> 8 weeks	6	University
	Corticosteroid injection in participants without perifascial oedema	Extracorporeal shockwave therapy		15	16						
Tiwari (2013) [57]	Corticosteroid injection	Platelet-rich plasma injection	NR	30	30	NR	NR	NR	NR	26	Hospital
Ugurlar (2018) [58]	Corticosteroid injection	Platelet-rich plasma injection	Acetaminophen for 3 days	40	39	50	38.8	26.9	> 52 weeks	156	Hospital
	Extracorporeal shockwave therapy	Prolotherapy		39	40						
Uygur (2018) [59]	Corticosteroid injection	Dry-needling	NR	47	49	66	49.6	NR	> 12 weeks	26	Hospital
Vahdatpour (2016) [60]	Corticosteroid injection	Platelet-rich plasma injection	Plantar fascia and calf stretches	16	16	72	46.2	29.6	> 12 weeks	26	Hospital
Whittaker (2019) [61]	Corticosteroid injection	Foot orthoses	Plantar fascia and calf stretches	50	53	61	43.9	30.4	> 4 weeks	12	University
Yesiltas (2015) [62]	Corticosteroid injection	Autologous blood injection	NR	21	28	57	45.5	30.4	NR	26	Hospital
Yucel (2010) [63]	Corticosteroid injection	Extracorporeal shockwave therapy	None permitted other than heel cups	33	27	70	43.9	NR	> 26 weeks	12	NR
Yucel (2013) [64]	Corticosteroid injection	Foot orthoses	Analgesia if required	20	20	80	46.4	30.1	> 12 weeks	4	University
Yuzer (2006) [65]	Corticosteroid injection	Laser therapy	NR	30	24	85	50.5	32.3	> 4 weeks	26	NR
Zamani (2014) [66]	Corticosteroid injection	Laser therapy	NR	20	20	57	52.5	NR	> 6 weeks	6	Rheumatology clinic

*Abbreviations*: *NR* Not reported, *BMI* Body mass index

^a^The minimum duration of symptoms that was specified in the inclusion criteria for the trial

**Table 2:** Characteristics of the corticosteroid injection used in each trial

Trial	Drug	Local anaesthetic	Ultrasound guidance	Needle placement
Abdihakin (2012) [20]	Methylprednisolone acetate	Lidocaine 1%	No	NR
Acosta-Olivo (2017) [21]	Dexamethasone isonicotinate	Lidocaine^a^	No	Point of maximal tenderness
Afsar (2015) [22]	NR	Lidocaine 1%	No	NR
Babaei-Ghazani (2019) [23]	Methylprednisolone acetate	Lidocaine 1%	Yes	Within the plantar fascia
Ball (2012) [24]	Methylprednisolone acetate	None. Skin anesthetized	Yes	Superficial to the plantar fascia enthesis
Celik (2015) [25]	Methylprednisolone acetate	Prilocaine 2%	No	Around the plantar fascia
Crawford (1999) [26]	Prednisolone acetate	Lidocaine 1%	No	Within flexor digitorum brevis
Diaz-Llopis (2012) [27]	Betamethasone acetate and betamethasone disodium phosphate	Mepivacaine 1%	No	Deep to quadratus plantae, near the plantar fascia insertion
Elizondo-Rodriguez (2013) [28]	Dexamethasone isonicotinate	Lidocaine 2%	No	Superior to the plantar fascia
Eslamian (2016) [29]	Methylprednisolone acetate	Lidocaine 2%	No	NR
Guevara Serna (2017) [30]	Methylprednisolone acetate	Lidocaine^a^	No	Point of maximal tenderness
Guner (2013) [31]	Methylprednisolone acetate	Lidocaine 2%	No	Peppering the plantar fascia
Hanselman (2015) [32]	Methylprednisolone acetate	Bupivacaine 0.5%	No	Inserted to calcaneal periosteum then ‘dragged’ across plantar fascia
Hocaoglu (2017) [33]	Betamethasone sodium phosphate	Prilocaine^a^	Yes	Into the thickest part of the plantar fascia, distal to its insertion on the calcaneus
Huo (2018) [34]	Betamethasone^a^	Lidocaine 2%	Yes	Within the thickest part of the plantar fascia
Jain (2015) [35]	Triamcinolone acetonide	Levobupivacaine^a^	No	Peppering the plantar fascia
Jain (2018) [36]	Methylprednisolone acetate	Lidocaine 2%	No	Point of maximal tenderness
Johannsen (2019) [37]	Methylprednisolone acetate	Lidocaine 1%	Yes	NR
Karimzadeh (2017) [38]	Methylprednisolone acetate	Lidocaine^a^	No	Point of maximal tenderness
Kiter (2006) [39]	Methylprednisolone acetate	Prilocaine 2%	No	NR
Kriss (2003) [40]	Triamcinolone hexacetonide	NR	No	NR
Lai (2018) [41]	Triamcinolone acetonide	Lidocaine 2%	No	NR
Lee (2007) [42]	Triamcinolone acetonide	Lidocaine 1%	No	Origin of the plantar fascia
Li (2014) [43]	Triamcinolone acetonide	Lidocaine 2%	No	Point of maximal tenderness
Mahindra (2016) [44]	Methylprednisolone acetate	NR	No	Peppering the plantar fascia
Mardani-Kivi (2015) [45]	Methylprednisolone acetate	Lidocaine 2%	No	Point of maximal tenderness
McMillan (2012) [46]	Dexamethasone sodium phosphate	Nil – provided tibial block	Yes	Within the plantar fascia
Monto (2014) [47]	Methylprednisolone acetate	Field block to the skin of bupivacaine 0.5%	Yes	NR
Mulherin (2009) [48]	Methylprednisolone^a^	Lidocaine 1%	No	Within the plantar fascia
Omar (2012) [49]	NR	NR	No	NR
Porter (2005) [50]	Betamethasone^a^	Lidocaine 1%	No	Point of maximal tenderness
Rastegar (2018) [51]	Methylprednisolone acetate	NR	No	Point of maximal tenderness
Ryan (2014) [52]	Dexamethasone^a^	Lidocaine 1%	No	Point of maximal tenderness
Saber (2012) [53]	Betamethasone diproprionate and betamethasone sodium phosphate	Lidocaine 0.5%	Yes	Within the plantar fascia
Serbest (2013) [54]	Betamethasone acetate and betamethasone sodium phosphate	Prilocaine 2%	No	Point of maximal tenderness
Shetty (2019) [55]	Methylprednisolone acetate	Lidocaine 1%	No	Peppering the point of maximal tenderness
Sorrentino (2008) [56]	Methylprednisolone acetate	Mepivacaine 3%	Yes	Within the plantar fascia
Tiwari (2013) [57]	Methylprednisolone acetate	Lidocaine 2%	No	Point of maximal tenderness
Ugurlar (2018) [58]	Betamethasone^a^	Bupivacaine 0.5%	Yes	Point of maximal tenderness
Uygur (2018) [59]	Methylprednisolone acetate	Bupivacaine 0.5%	No	Between the plantar fascia and the periosteum, with peppering
Vahdatpour (2016) [60]	Methylprednisolone acetate	Lidocaine^a^	No	Point of maximal tenderness
Whittaker (2019) [61]	Betamethasone acetate and betamethasone sodium phosphate	Bupivacaine 0.5%	Yes	Deep and superficial to the plantar fascia
Yesiltas (2015) [62]	Triamcinolone^a^ (mixed with distilled water)	NR	No	NR
Yucel (2010) [63]	Betamethasone diproprionate and betamethasone sodium phosphate	Prilocaine 2%	No	Point of maximal tenderness
Yucel (2013) [64]	Betamethasone diproprionate and betamethasone sodium phosphate	Lidocaine^a^	Yes	Within the plantar fascia
Yuzer (2006) [65]	Betamethasone diproprionate and betamethasone sodium phosphate	Prilocaine 2%	No	Point of maximal tenderness
Zamani (2014) [66]	Methylprednisolone acetate	NR	No	Point of maximal tenderness

*Abbreviations*: *NR* Not reported

^a^No other information provided

**Fig. 2:**
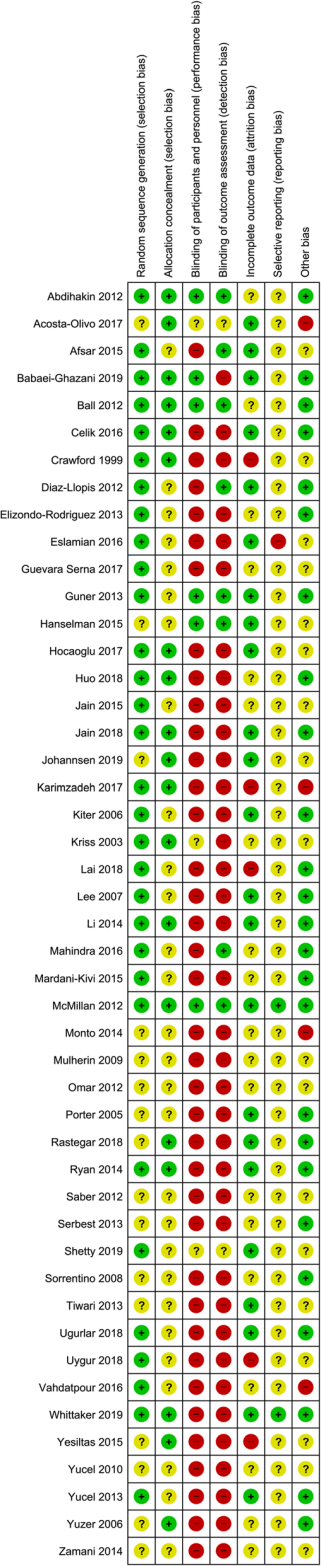
Risk of bias summary for each included trial

Risk of bias assessment (Fig. 2) revealed that 1/47 of the included trials was low risk, 41/47 were high risk, and 5/47 were of unclear risk. A frequent contributor (39/47 trials) to high risk of bias was not blinding participants/personnel and outcome assessors. There was a moderate [18] level of agreement between the authors (SEM and DRB) who assessed risk of bias (*κ* = 0.46; 95% CI, 0.40 to 0.50, *P* < 0.001).

GRADE evidence profiles are presented in Tables 3 and 4. Ratings were made at short, medium and longer term-time points for comparisons that had sufficient data to conduct meta-analyses. Ratings were only made for the primary outcomes of pain and function as they were considered the most important outcomes for patients [68].

**Table 3:** GRADE evidence profile of the effect of corticosteroid injection on pain

Quality assessment							Summary of findings			
Comparison	No. of trials	Limitations	Inconsistency	Indirectness	Imprecision	Publication bias	Participants		Effect size (95% CI)^a^	GRADE
							Corticosteroid injection	Comparator		
Corticosteroid injection vs placebo injection										
Short term	4 [20, 24, 44, 46]	No serious limitations	Serious inconsistency^b^	No serious indirectness	Serious imprecision^c^	Undetected	132	123	-0.98 (−2.06, 0.11)^f^	Moderate
Medium term	4 [20, 24, 44, 46]	No serious limitations	Serious inconsistency^b^	No serious indirectness	Serious imprecision^c^	Undetected	126	122	-0.86 (− 1.90, 0.19)^f^	Moderate
Corticosteroid injection vs physical therapy										
Short term	2 [25, 52]	Very serious limitations^d^	Serious inconsistency^b^	No serious indirectness	Serious imprecision^e^	Undetected	49	50	-1.07 (−2.75, 0.60)^f^	Very low
Medium term	3 [25, 37, 52]	Very serious limitations^d^	Serious inconsistency^b^	No serious indirectness	No serious imprecision	Undetected	80	79	-0.74 (− 1.51, 0.03)^f^	Low
Longer term	2 [25, 37]	Very serious limitations^d^	No serious inconsistency	No serious indirectness	Serious imprecision^c^	Undetected	52	51	0.00 (−0.39, 0.38)	Very low
Corticosteroid injection vs foot orthoses										
Short term	3 [40, 61, 64]	Very serious limitations^d^	Serious inconsistency^b^	No serious indirectness	No serious imprecision	Undetected	92	99	−0.91 (−1.69, − 0.13)^f^	Low
Medium term	3 [40, 61, 64]	Very serious limitations^d^	Serious inconsistency^b^	Serious indirectness^g^	Serious imprecision^c^	Undetected	72	79	−0.17 (− 1.30, 0.97)	Very low
Corticosteroid injection vs dry needling										
Short term	2 [51, 59]	Very serious limitations^d^	Very serious inconsistency^b^	No serious indirectness	Serious imprecision^c^	Undetected	81	81	−0.86 (−3.70, 1.97)^f^	Very low
Longer term	2 [51, 59]	Very serious limitations^d^	Serious inconsistency^b^	No serious indirectness	No serious imprecision	Undetected	81	81	1.45 (0.70, 2.19)^f^	Low
Corticosteroid injection vs extracorporeal shockwave therapy										
Short term	8 [29, 33, 34, 41, 45, 54, 56, 58]	Very serious limitations^d^	Serious inconsistency^b^	No serious indirectness	No serious imprecision	Undetected	269	265	−0.32 (−0.77, 0.12)	Very low
Medium term	10 [29, 30, 33, 34, 41, 45, 50, 54, 58, 63]	Very serious limitations^d^	Serious inconsistency^b^	No serious indirectness	Serious imprecision^c^	Undetected	354	354	−0.05 (−0.60, 0.49)	Very low
Longer term	5 [30, 33, 34, 50, 58]	Very serious limitations^d^	Serious inconsistency^b^	No serious indirectness	No serious imprecision	Undetected	202	211	0.45 (−0.09, 0.99)	Very low
Corticosteroid injection vs laser therapy										
Short term	2 [65, 66]	Very serious limitations^d^	No serious inconsistency	Serious indirectness^g^	Serious imprecision^c^	Undetected	50	44	−0.20 (−0.61, 0.20)	Very low
Corticosteroid injection vs autologous blood injection										
Short term	4 [22, 38, 42, 62]	Very serious limitations^d^	No serious inconsistency	No serious indirectness	No serious imprecision	Undetected	126	131	−0.56 (−0.86, − 0.26)	Low
Medium term	4 [22, 38, 42, 62]	Very serious limitations^d^	Serious inconsistency^b^	No serious indirectness	Serious imprecision^c^	Undetected	126	131	−0.31 (−0.83, 0.21)	Very low
Longer term	4 [22, 39, 42, 62]	Very serious limitations^d^	No serious inconsistency	No serious indirectness	Serious imprecision^c^	Undetected	128	134	−0.05 (−0.31, 0.21)	Very low
Corticosteroid injection vs platelet-rich plasma injection										
Short term	8 [21, 35, 36, 44, 49, 57, 58, 60]	Very serious limitations^d^	Serious inconsistency^b^	No serious indirectness	Serious imprecision^c^	Undetected	202	203	−0.16 (−0.70, 0.38)	Very low
Medium term	7 [21, 35, 36, 44, 57, 58, 60]	Very serious limitations^d^	Serious inconsistency^b^	No serious indirectness	Serious imprecision^c^	Undetected	187	188	0.32 (−0.19, 0.83)	Very low
Longer term	6 [21, 35, 36, 57, 58, 60]	Very serious limitations^d^	Serious inconsistency^b^	Serious indirectness^h^	No serious imprecision	Undetected	162	163	0.61 (0.30, 1.06)	Very low
Corticosteroid injection vs botulinum toxin-A injection										
Short term	2 [27, 28]	Very serious limitations^d^	Serious inconsistency^b^	No serious indirectness	Serious imprecision^e^	Undetected	45	45	0.67 (−0.04, 1.38)	Very low

*Abbreviations*: *CI* Confidence interval, *GRADE* Grading Recommendations Assessment, Development and Evaluation

^a^ Negative values indicate that the effect size (SMD) favours corticosteroid injection

^b^ Rated down 1 level for consistency as there was significant heterogeneity (i.e. *I*^2^ greater than 40%)

^c^ Rated down 1 level as the upper and lower boundaries of the confidence intervals represent different conclusions

^d^ All participants for this outcome were from trials rated at high risk of bias

^e^ The total sample for this outcome is less than 100

^f^ Rated up 1 level due to large effect size

^g^ The interventions differed between studies

^h^ Outcome measures were obtained at significantly different time points

**Table 4:** GRADE evidence profile of the effect of corticosteroid injection on function

Quality assessment							Summary of findings			
Comparison	No. of trials	Limitations	Inconsistency	Indirectness	Imprecision	Publication bias	Participants		Effect size (95% CI)^a^	GRADE
							Corticosteroid injection	Comparator		
Corticosteroid injection vs physical therapy										
Short term	2 [25, 52]	Very serious limitations^b^	Serious inconsistency^c^	No serious indirectness	No serious imprecision	Undetected	49	50	−0.69 (−1.31, − 0.07)	Low
Medium term	2 [25, 52]	Very serious limitations^b^	Serious inconsistency^c^	No serious indirectness	Serious imprecision^d^	Undetected	49	50	−0.55 (− 1.14, 0.03)	Very low
Corticosteroid injection vs foot orthoses										
Short term	2 [61, 64]	Very serious limitations^b^	Serious inconsistency^c^	No serious indirectness	Serious imprecision^d^	Undetected	70	73	−0.78 (−1.81, 0.25)	Very low
Corticosteroid injection vs extracorporeal shockwave therapy										
Short term	2 [41, 58]	Very serious limitations^b^	No serious inconsistency	No serious indirectness	Serious imprecision^d^	Undetected	90	86	0.11 (−0.18, 0.41)	Very low
Medium term	2 [41, 58]	Very serious limitations^b^	No serious inconsistency	No serious indirectness	Serious imprecision^d^	Undetected	90	86	0.21 (−0.08, 0.51)	Very low
Corticosteroid injection vs platelet-rich plasma injection										
Short term	3 [21, 36, 58]	Very serious limitations^b^	No serious inconsistency	No serious indirectness	No serious imprecision	Undetected	94	93	−0.18 (−0.47, 0.10)	Low
Medium term	3 [21, 36, 58]	Very serious limitations^b^	No serious inconsistency	No serious indirectness	Serious imprecision^d^	Undetected	94	93	0.10(−0.18, 0.39)	Very low
Longer term	3 [21, 36, 58]	Very serious limitations^b^	No serious inconsistency	No serious indirectness	No serious imprecision	Undetected	94	93	0.21 (−0.08, 0.49)	Low
Corticosteroid injection vs botulinum toxin-A injection										
Short term	2 [27, 28]	Very serious limitations^b^	Serious inconsistency^c^	No serious indirectness	Serious imprecision^d^	Undetected	45	45	0.76 (−0.24, 1.76)	Very low

*Abbreviations*: *CI* Confidence interval, *GRADE* Grading Recommendations Assessment, Development and Evaluation

^a^ Negative values indicate that the effect size (SMD) favours corticosteroid injection

^b^ All participants for this outcome were from trials rated at high risk of bias

^c^ Rated down 1 level for consistency as there was significant heterogeneity (i.e. *I*^2^ greater than 40%)

^d^ Rated down 1 level as the upper and lower boundaries of the confidence intervals represent different conclusions

### Primary outcomes

#### Pain

Results of trials that could not be pooled in meta-analyses are summarised in Additional file 3. Pooled point estimates with negative values indicate an effect in favour of corticosteroid injection.

**Fig. 3:**
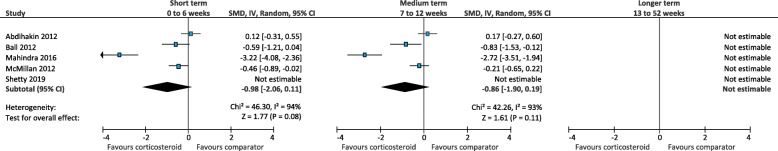
Meta-analyses comparing corticosteroid injection to placebo injection for the outcome of pain. *SMD = standard mean difference; IV = inverse variance; CI = confidence interval*

Data for the comparison of corticosteroid injection to placebo injection were available from four trials [20, 24, 44, 46] in the short and medium terms, and no data were available in the longer term (Fig. 3). There was moderate quality evidence that corticosteroid injection is similar to placebo injection in the short (SMD -0.98; 95% CI, − 2.06 to 0.11) and medium terms (SMD -0.86; 95% CI, − 1.90 to 0.19).

**Fig. 4:**
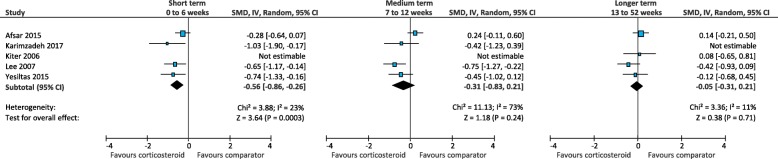
Meta-analyses comparing corticosteroid injection to autologous blood injection for the outcome of pain. *SMD = standard mean difference; IV = inverse variance; CI = confidence interval*

**Fig. 5:**
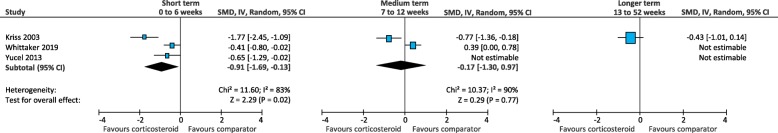
Meta-analyses comparing corticosteroid injection to foot orthoses for the outcome of pain. *SMD = standard mean difference; IV = inverse variance; CI = confidence interval*

**Fig. 6:**
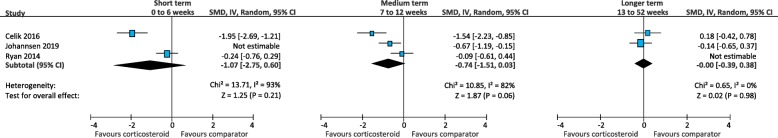
Meta-analyses comparing corticosteroid injection to physical therapy for the outcome of pain. *SMD = standard mean difference; IV = inverse variance; CI = confidence interval*

**Fig. 7:**
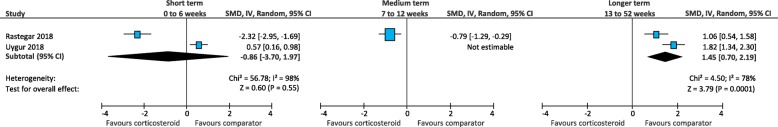
Meta-analyses comparing corticosteroid injection to dry needling for the outcome of pain. *SMD = standard mean difference; IV = inverse variance; CI = confidence interval*

**Fig. 8:**
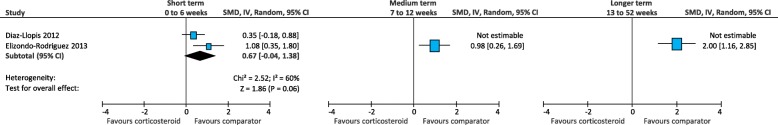
Meta-analyses comparing corticosteroid injection to botulinum toxin-A injection for the outcome of pain. *SMD = standard mean difference; IV = inverse variance; CI = confidence interval*

**Fig. 9:**
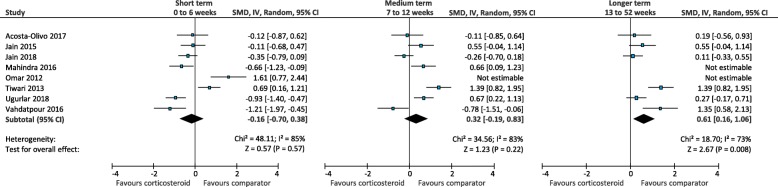
Meta-analyses comparing corticosteroid injection to platelet-rich plasma injection for the outcome of pain. *SMD = standard mean difference; IV = inverse variance; CI = confidence interval*

**Fig. 10:**
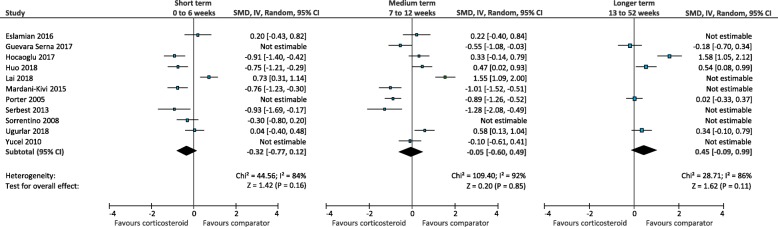
Meta-analyses comparing corticosteroid injection to extracorporeal shockwave therapy for the outcome of pain. *SMD = standard mean difference; IV = inverse variance; CI = confidence interval*

**Fig. 11:**
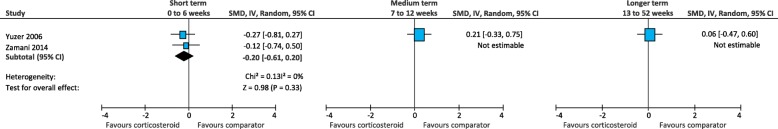
Meta-analyses comparing corticosteroid injection to laser therapy for the outcome of pain. *SMD = standard mean difference; IV = inverse variance; CI = confidence interval*

**Fig. 12:**
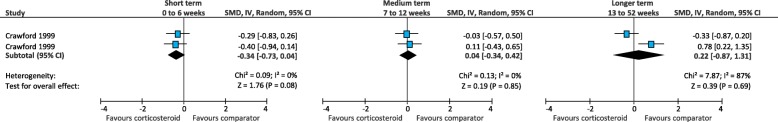
Meta-analyses comparing corticosteroid injection to local anaesthetic injection for the outcome of pain. *SMD = standard mean difference; IV = inverse variance; CI = confidence interval*

When corticosteroid injection was compared to other comparators in the short term (0 to 6 weeks), there was low quality evidence that corticosteroid injection is more effective than autologous blood injection (SMD -0.56; 95% CI, − 0.86 to − 0.26) (Fig. 4) [22, 38, 42, 62] and foot orthoses (SMD -0.91; 95% CI, − 1.69 to − 0.13) (Fig. 5) [40, 61, 64]. There was very-low quality evidence that corticosteroid injection is similar to physical therapy (SMD -1.07; 95% CI, − 2.75 to 0.60) (Fig. 6) [25, 52], dry needling (SMD -0.86; 95% CI, − 3.70 to 1.97) (Fig. 7) [51, 59], botulinum toxin-A injection (SMD 0.67; 95% CI, − 0.04 to 1.38) (Fig. 8) [27, 28], platelet-rich plasma injection (SMD -0.16; 95% CI, − 0.70 to 0.38) (Fig. 9) [21, 35, 36, 44, 49, 57, 58, 60], extracorporeal shockwave therapy (SMD -0.32; 95% CI, − 0.77 to 0.12) (Fig. 10) [29, 33, 34, 41, 45, 54, 56, 58], laser therapy (SMD -0.20; 95% CI, − 0.61 to 0.20) (Fig. 11) [65, 66], and local anaesthetic injection (SMD -0.34; 95% CI, − 0.73 to 0.04) (Fig. 12) [26].

In the medium term (7 to 12 weeks), there was low quality evidence that corticosteroid injection is similar to physical therapy (SMD -0.74; 95% CI, − 1.51 to 0.03) [25, 37, 52], and very-low quality evidence corticosteroid injection is similar to autologous blood injection (SMD -0.31; 95% CI, − 0.83 to 0.21) [22, 38, 42, 62], foot orthoses (SMD -0.17; 95% CI; − 1.30 to 0.97) [40, 61], platelet-rich plasma injection (SMD 0.32; 95% CI, − 0.19 to 0.83) [21, 35, 36, 44, 57, 58, 60], extracorporeal shockwave therapy (SMD -0.05; 95% CI, − 0.60 to 0.49) [29, 30, 33, 34, 41, 45, 50, 54, 58, 63], and local anaesthetic injection (SMD 0.04; 95% CI, − 0.34 to 0.42) [26].

In the longer term (13 to 52 weeks), there was low quality evidence that corticosteroid injection is less effective than dry needling (SMD 1.45; 95% CI, 0.70 to 2.19) [51, 59], and very low-quality evidence corticosteroid injection is less effective than platelet-rich plasma injection (SMD 0.61; 95% CI, 0.16 to 1.06) [21, 35, 36, 57, 58, 60]. There was very-low quality evidence that corticosteroid injection is similar to physical therapy (SMD -0.00; 95% CI − 0.39 to 0.38) [25, 37] autologous blood injection (SMD -0.05; 95% CI, − 0.31 to 0.21) [22, 39, 42, 62], extracorporeal shockwave therapy (SMD 0.45; 95% CI, − 0.09 to 0.99) [30, 33, 34, 50, 58], and local anaesthetic injection (SMD 0.22; 95% CI, − 0.87 to 1.31) [26].

**Fig. 13:**
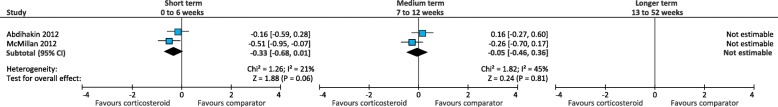
Meta-analyses comparing corticosteroid injection to placebo injection for the outcome of ‘first step’ pain. *SMD = standard mean difference; IV = inverse variance; CI = confidence interval*

For ‘first-step’ pain, meta-analyses were possible for trials that compared corticosteroid injection to placebo injection in the short and medium terms (Fig. 13). Corticosteroid injection was similar to placebo injection in the short (SMD -0.33; 95% CI, − 0.68 to 0.01) and medium terms (SMD -0.05; 95% CI, − 0.46 to 0.36) [20, 46]. Results from trials that could not be pooled in meta-analyses are summarised in Additional file 4.

#### Function

**Fig. 14:**
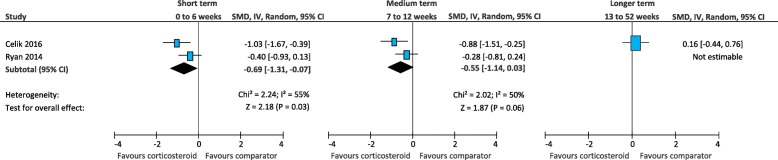
Meta-analyses comparing corticosteroid injection to physical therapy for the outcome of function. *SMD = standard mean difference; IV = inverse variance; CI = confidence interval*

**Fig. 15:**
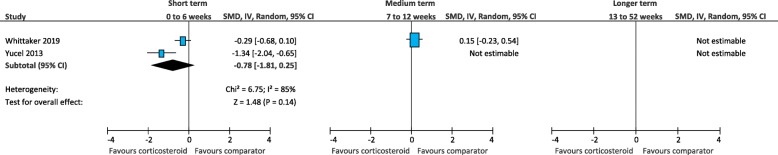
Meta-analyses comparing corticosteroid injection to foot orthoses for the outcome of function. *SMD = standard mean difference; IV = inverse variance; CI = confidence interval*

**Fig. 16:**
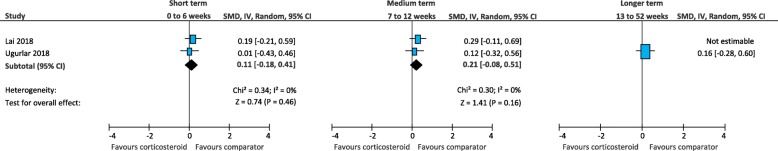
Meta-analyses comparing corticosteroid injection to extracorporeal shockwave therapy for the outcome of function. *SMD = standard mean difference; IV = inverse variance; CI = confidence interval*

**Fig. 17:**
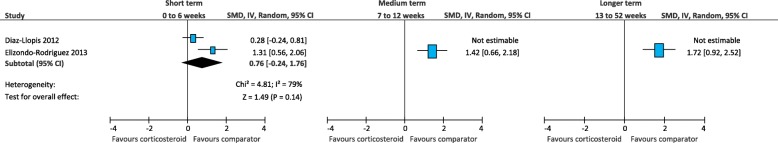
Meta-analyses comparing corticosteroid injection to botulinum toxin-A injection for the outcome of function. *SMD = standard mean difference; IV = inverse variance; CI = confidence interval*

**Fig. 18:**
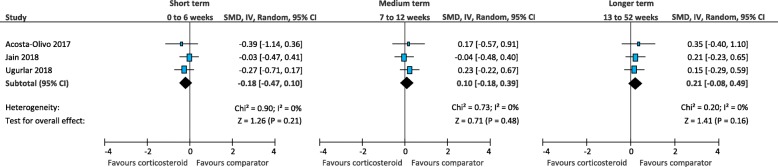
Meta-analyses comparing corticosteroid injection to platelet-rich plasma injection for the outcome of function. *SMD = standard mean difference; IV = inverse variance; CI = confidence interval*

In the short term, there was low quality evidence that corticosteroid injection is more effective than physical therapy (SMD -0.69; 95% CI, − 1.31 to − 0.07) (Fig. 14) [25, 52]. There was very-low quality evidence that corticosteroid injection is similar to foot orthoses (SMD -0.78; 95% CI, − 1.81 to 0.25) (Fig. 15) [61, 64], extracorporeal shockwave therapy (SMD 0.11; 95% CI, − 0.18 to 0.41) (Fig. 16) [41, 58], and botulinum toxin-A injection (SMD 0.76; 95% CI, − 0.24 to 1.76) (Fig. 17) [27, 28]. There was low quality evidence that corticosteroid injection is similar to platelet-rich plasma injection (SMD -0.18; 95% CI − 0.47 to 0.10) (Fig. 18) [21, 36, 58],

In the medium term, there was very-low quality evidence that corticosteroid injection is similar to physical therapy (SMD -0.55; 95% CI, − 1.14 to 0.03) [25, 52], extracorporeal shockwave therapy (SMD 0.21; 95% CI − 0.08 to 0.51) [41, 58], and platelet-rich plasma injection (SMD 0.10; 95% CI, − 0.18 to 0.39) [21, 36, 58].

In the longer term, there was low quality evidence that corticosteroid injection is similar to platelet-rich plasma injection (SMD 0.21; 95% CI, − 0.08 to 0.49) [21, 36, 58]. Results of trials that could not be pooled in meta-analyses are summarised in Additional file 5.

### Secondary outcomes

#### Plantar fascia thickness

**Fig. 19:**
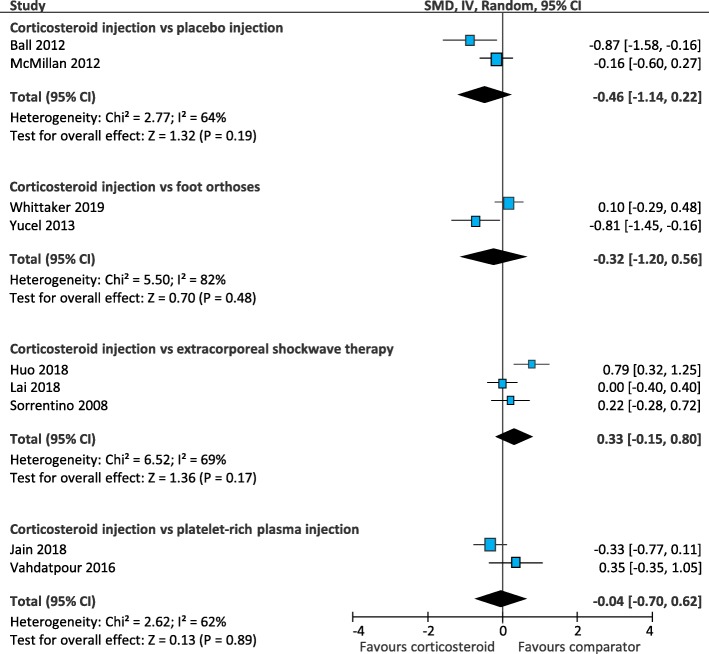
Meta-analyses for the outcome of plantar fascia thickness. *SMD = standard mean difference; IV = inverse variance; CI = confidence interval*

Values extracted for plantar fascia thickness were from the last time point reported in each trial. Corticosteroid injection was similar to placebo injection (SMD -0.46; 95% CI, − 1.14 to 0.22) [24, 46], foot orthoses (SMD-0.32; 95% CI − 1.20 to 0.56) [61, 64], extracorporeal shockwave therapy (SMD 0.33; 95% CI, − 0.15 to 0.80) [34, 41, 56], and platelet-rich plasma injection (SMD -0.04; 95% CI, − 0.70 to 0.62) [36, 60] (Fig. 19). Results from trials that could not be pooled in meta-analyses are summarised in Additional file 6.

### Sensitivity analysis

A sensitivity analysis was conducted that excluded trials considered to have high risk of bias. For pain, there was sufficient data for meta-analysis from three trials [20, 24, 46], which found corticosteroid injection is similar to placebo injection in the short (SMD -0.28; 95% CI, − 0.71 to 0.16) and medium terms (SMD -0.23; 95% CI, − 0.72 to 0.28). No data were available for meta-analysis from other comparators. The findings for ‘first step’ pain were unchanged with the sensitivity analysis. For function, no data were available, so a sensitivity analysis was not conducted. Finally, the findings for the secondary outcome measure of plantar fascia thickness were unchanged with sensitivity analysis for the comparison to placebo injection only.

### Adverse events

Adverse events were assessed in 30/47 trials [21–24, 27–32, 34–38, 40, 42, 43, 46, 50, 55–59, 61–65]. In 25 of the 30 trials where adverse events were assessed [21, 22, 24, 25, 27–32, 35, 40, 43, 46, 53, 56, 57, 62–65], no adverse events were reported. In the remaining 5 trials, the only adverse event that was reported was post-injection pain [37, 38, 42, 50, 63].

## Discussion

The findings of this systematic review indicate that for the outcome of pain, corticosteroid injection is more effective than autologous blood injection and foot orthoses in the short term (up to 6 weeks), but platelet-rich plasma and dry needling are more effective in the longer term (greater than 12 weeks). For the outcome of function, corticosteroid injection is more effective than physical therapy in the short term. Notably, corticosteroid injection is similar to placebo injection for pain and function.

The finding that corticosteroid injection is similar to placebo injection for the outcome of pain is notable. Many health professionals would perceive a discordance between this finding and reductions in pain observed in clinical practice following corticosteroid injection. However, this may be explained by *non-specific* effects from influences such as natural resolution, regression to the mean, the placebo effect, or expectancy effects [69, 70]. These non-specific effects cannot be disregarded and our findings may suggest that any *specific* effect from the corticosteroid drug itself is small. Indeed, in similar work relating to knee osteoarthritis, non-specific effects account for almost half of the overall effect observed for corticosteroid injection [71].

For comparators other than placebo injection, we found corticosteroid injection to be more effective for the reduction of pain than autologous blood injection and foot orthoses in the short term. Although meta-analyses for the remaining comparators in the short term were not statistically significant, there was a general trend for corticosteroid injection to be more effective (based on meaningful effect sizes). However, this trend diminished in the medium to longer term. Statistically significant findings, with moderate to large effect sizes, were found for the comparison to dry needling (SMD of 1.45) and platelet-rich plasma injection (SMD of 0.61). Therefore, compared to the variety of other comparators included in this review, corticosteroid injection is more effective compared to comparators in the short term but not in the longer term. Further research will improve the precision of these estimates and the conclusions that can be drawn, especially regarding the effectiveness of corticosteroid injection in the short term.

For ‘first-step’ pain, few trials reported this outcome and a meta-analysis was only possible for the comparison between corticosteroid injection and placebo injection, which found that corticosteroid injection was similar to placebo injection in the short term. However, this finding was close to being statistically significant with the upper confidence limit just including zero (SMD -0.33; 95% CI, − 0.68 to 0.01). This finding remained unchanged after excluding trials considered to have a high risk of bias. Given ‘first step’ pain is a principal complaint of patients with plantar heel pain, it is important that future clinical trials evaluate ‘first step’ pain as an outcome.

There were few trials that reported function as an outcome, and meta-analyses were only possible for comparisons to physical therapy, foot orthoses, extracorporeal shockwave therapy, platelet-rich plasma injection, and botulinum toxin-A injection. The only significant finding was for the comparison between corticosteroid injection and physical therapy, which found corticosteroid injection to be more effective in the short term. Single trials, and meta-analyses that were not significantly different, tended to find corticosteroid injection was more effective in the short term, but the comparator intervention was found to be more effective in the medium and longer term.

We also investigated the secondary outcome of plantar fascia thickness – a biological outcome rather than a patient-reported outcome. Meta-analyses found corticosteroid injection was not more effective than other comparators for the reduction of plantar fascia thickness. However, there was a trend for corticosteroid injection to be more effective than placebo injection and for extracorporeal shockwave therapy to be more effective than corticosteroid injection. It is important to note, however, that because this was a secondary outcome, it was not included in our original search strategy, so there is a small chance that additional trials that measured this outcome may have been missed.

The findings above should be interpreted with regard to the quality of the trials that investigated the effectiveness of corticosteroid injection. According to GRADE, the findings of these studies ranged from *very-low* to *moderate* quality, which means we have limited confidence in the findings and they are likely to change when future trials are conducted. Furthermore, most trials (39/47) were at high risk of bias, and when a sensitivity analysis was performed that excluded these trials, there were no significant findings.

### Clinical importance

To provide a sense of the clinical worth of these findings, statistically significant results for pain were back-transformed to a 0–100 point visual analogue scale [14], and compared to the previously calculated minimal important difference value of 8 points (on a 0–100 point scale) [72] using a pooled standard deviation [15]. Although this method provides a sense of whether the difference between these interventions is clinically worthwhile, these estimates can be misleading and should be interpreted with caution [73]. In the short term, corticosteroid injection provided a clinically worthwhile effect when compared to foot orthoses (between-group difference of 12.2 points) and autologous blood injection (between-group difference of 14.8 points). In the longer term, dry needling (between-group difference of 18.9 points) and platelet-rich plasma injection (between-group difference of 10.0 points) provided a clinically worthwhile effect when compared to corticosteroid injection. For function, the clinical worth of corticosteroid injection compared to physical therapy could not be estimated as the minimal important difference values have not been calculated for the outcome measures used by trials in that meta-analysis.

Importantly, these findings were all from trials at high risk of bias, which may exaggerate clinical effectiveness. An example of the influence of bias is the comparison between corticosteroid injection and placebo injection in the short term. After excluding trials at high risk of bias, the estimate of the clinical importance of this comparison (although not statistically significant) reduced from 18.0 points to 4.7 points (on a 0–100 point scale). This reduction should be noted by health professionals, and it reiterates our earlier comment that non-specific effects may influence the reporting of pain.

### Limitations and directions for future research

There was substantial heterogeneity (as indicated by the high *I*^*2*^ values) for most meta-analyses conducted, and this may reflect several recurring methodological issues. First, there were a variety of corticosteroids, combined anaesthetics, injection techniques, and comparators used in the included trials. Second, the mean group size for trials was 28 participants, and most trials did not report a priori sample size calculations. Finally, there was a lack of participant and investigator blinding, which was a common reason that trials were considered to have a high risk of bias. For trials with interventions such as physical therapy, it is almost impossible to blind the participant, however for injectable therapeutic solutions (e.g. autologous blood or platelet-rich plasma), it is possible to achieve participant and investigator blinding [74]. With these shortcomings in mind, the strength of the overall body of evidence is reduced and the recommendations that can be made are limited.

We found that corticosteroid injection was a safe intervention, with post-injection pain the only reported adverse effect. Two case-series studies published in the 1990s suggested there may be an increased risk of plantar fascia rupture following corticosteroid injection [75, 76], although no plantar fascia ruptures have been reported for participants who received a corticosteroid injection in the randomised trials included in our review. Long-term adverse effects of a corticosteroid injection are unclear, as few trials reported outcomes beyond 12 weeks. This is an important consideration as there are reports that corticosteroid injection has a deleterious long-term effect on tendon [77], and one trial that followed participants with lateral epicondylitis for 1 year found that the group that received a corticosteroid injection had more pain than a ‘wait and see’ group at the conclusion of the trial [78]. Worryingly, some trials [20, 26, 33, 39, 41, 44, 45, 47–49, 51–54, 60] included in our review did not report adverse events, and few reported whether they actively questioned participants about adverse events.

## Conclusions

For the outcome of pain in the short term, we found *low quality* evidence that corticosteroid injection is more effective than autologous blood injection and foot orthoses. In the longer term, we found *very-low quality* evidence that corticosteroid injection is less effective than dry needling and platelet-rich plasma injection. These findings were greater than minimal important difference values, indicating that they are clinically worthwhile. For the outcome of function, we found *low quality* evidence that corticosteroid injection is more effective than physical therapy, but this was only in the short term. Notably, corticosteroid injection was found to have similar effectiveness to placebo injection for pain and function. The impact of bias on these findings was assessed with a sensitivity analysis, which found that corticosteroid injection had similar effectiveness to placebo injection. Further trials that are of low risk of bias will strengthen this evidence.

## Additional files

Additional file 1: Search strategy. The search strategy used for the systematic search. (PDF 61 kb)

Additional file 2: Criteria used for judgements of GRADE. (PDF 64 kb)

Additional file 3: Results of single trials that investigated pain. A summary of the findings from single trials that investigated pain but were not included in a meta-analysis. (PDF 95 kb)

Additional file 4: Results of single trials that investigated ‘first step’ pain. A summary of the findings from single trials that investigated ‘first step’ pain but were not included in a meta-analysis. (PDF 109 kb)

Additional file 5: Results of single trials that investigated function. A summary of the findings from single trials that investigated function but were not included in a meta-analysis. (PDF 82 kb)

Additional file 6: Results of single trials that investigated plantar fascia thickness. A summary of the findings from single trials that investigated plantar fascia thickness but were not included in a meta-analysis. (PDF 65 kb)

## Data Availability

The dataset supporting the conclusions of this article is available in the figshare repository: Whittaker, Glen; Munteanu, Shannon; Menz, Hylton; Bonanno, Daniel; Gerrard, James; Landorf, Karl (2018): Extracted data for a systematic review and meta-analyses of corticosteroid injection for plantar heel pain. Figshare. Dataset. 10.4225/22/5afa8ad63d93d

## Abbreviations

BMI: Body mass index
CI: Confidence interval
GRADE: Grading of Recommendations, Assessment, Development and Evaluation
PRISMA: Preferred Reporting Items for Systematic Reviews and Meta-analyses
SMD: Standardised mean difference
