# Supplementary material for: Corticosteroid injection for plantar heel pain: a systematic review and meta-analysis
Source: BMC Musculoskelet Disord. 2019 Aug 17;20:378. doi: 10.1186/s12891-019-2749-z (PMC6698340; doi:10.1186/s12891-019-2749-z)
Supplement: Supplementary file 1 — Search strategy. The search strategy used for the systematic search. (PDF 61 kb) [file 12891_2019_2749_MOESM1_ESM.pdf]

Additional file 1. Search strategy.

MEDLINE and EMBASE (Ovid)	CINAHL (EBSCO)	Cochrane Library
1. exp ADRENAL CORTEX HORMONES/	1. MH “Adrenal Cortex Hormones+”	1. MeSH Adrenal Cortex Hormones exp
2. exp STEROIDS/	2. MH “Injections+”	2. MeSH Injections exp
3. exp INJECTIONS/	3. MH “Antiinflammatory Agents, Steroidal+”	3. MeSH Steroids exp
4. corticosteroid* or injection*	4. corticosteroid* or injection*	4. corticosteroid* or injection*
5. #1 or #2 or #3 or #4	5. #1 or #2 or #3 #4	5. #1 or #2 or #3 or #4
6. exp FASCIITIS, PLANTAR/	6. MH “plantar fasciitis+”	6. MeSH Fasciitis, Plantar exp
7. exp HEEL SPUR/	7. MH “foot diseases+”	7. MeSH Heel Spur exp
8. PLANTAR FASCI*	8. MH “heel spur+”	8. “plantar fasci*”
9. (PLANTAR or HEEL or CALCANE*) adj2 PAIN*	9. MH calcaneus+	9. (plantar or heel or cancale*) near pain*
10. SPUR* or ENTHES* or PERIOSTITIS) adj2 CALCANE*	10. Plantar fasci*	10. (spur* or enthes* or periostitis*) near calcane*
11. #6 or #7 or #8 or #9 or #10	11. (plantar or heel or calcane* or foot) N2 pain*	11. #6 or #7 or #8 or #9 or #10
12. #5 and #11	12. (spur* or enthes* or periostitis) N2 calcane*	12. #5 and #11
13. Limit #12 to Human	13. #6 or #7 or #8 or #9 or #10 or #11 or # 12	
	14. #5 and #13	
	15. Limit #14 to Human	

Abbreviations: exp, explode; MH, major heading; MeSH, medical subject headings.
