# Supplementary material for: Corticosteroid injection for plantar heel pain: a systematic review and meta-analysis
Source: BMC Musculoskelet Disord. 2019 Aug 17;20:378. doi: 10.1186/s12891-019-2749-z (PMC6698340; doi:10.1186/s12891-019-2749-z)
Supplement: Supplementary file 2 — Criteria used for judgements of GRADE. (PDF 64 kb) [file 12891_2019_2749_MOESM2_ESM.pdf]

Additional file 2. Criteria used for judgments of GRADE.

Effect sizes were back-transformed from standard mean differences to the 100 mm visual analogue scale. Outcomes were rated down one level for *limitations* if greater than 25% of the participants included in the outcome came from studies considered to have a high risk of bias. Studies were rated down one level for *inconsistency* if there was significant heterogeneity (i.e. *I2* greater than 40%) [1]. Outcomes were rated down for *indirectness* if there were significant differences between the populations, interventions or outcomes measured across studies [2]. Outcomes were rated down for *imprecision* if the confidence intervals represented different conclusions and/or the total participants included for the outcome was less than 100. This sample size was calculated using the minimal important difference of 12 points on the 100 point Foot Health Status Questionnaire pain subscale,[3] and a standard deviation of 21 derived from previous research [4,5]. This provides 80% power to detect a moderate standard mean difference of 0.5 [6]. *Publication bias* was assessed using funnel plots for analyses with greater than 10 studies or if there was obvious industry involvement [1]. Outcomes for each comparison were classified into four categories: (i) *high* (we are very confident that the true effect lies close to the estimate of effect), (ii) *moderate* (we are moderately confident in the effect estimate: the true effect is likely to be close to the estimate of the effect but there is a possibility that it is substantially different), (iii) *low* (our confidence in the effect estimate is limited: the true effect may be substantially different from the estimate of effect), and (iv) *very low* (we have very little confidence in the effect estimate: the true effect is likely to be substantially different from the estimate of effect) [7].

References

1 Higgins JP, Green S. *Cochrane Handbook for Systematic Reviews of Interventions*. Wiley Online Library 2008. doi:10.1002/9780470712184

2 Guyatt GH, Oxman AD, Kunz R, *et al.* GRADE guidelines: 8. Rating the quality of evidence—indirectness. *J Clin Epidemiol* 2011;**64**:1303–10. doi:10.1016/j.jclinepi.2011.04.014

3 Landorf KB, Radford JA, Hudson S. Minimal Important Difference (MID) of two commonly used outcome measures for foot problems. *J Foot Ankle Res* 2010;**3**:7. doi:10.1186/1757-1146-3-7

4 Landorf KB, Keenan A-M, Herbert RD. Effectiveness of foot orthoses to treat plantar fasciitis. *Arch Intern Med* 2006;**166**:1305–10. doi:10.1001/archinte.166.12.1305

5 McMillan AM, Landorf KB, Gilheany MF, *et al.* Ultrasound guided corticosteroid injection for plantar fasciitis: randomised controlled trial. *BMJ* 2012;**344**:e3260. doi:10.1136/bmj.e3260

6 Cohen J. *Statistical Power Analysis for the Behavioral Sciences*. 2nd ed. Hillsdale, HJ: Lawrence Erlbaum Associates 1988.

7 Balshem H, Helfand M, Schünemann HJ, *et al.* GRADE guidelines: 3. Rating the quality of evidence. *J Clin Epidemiol* 2011;**64**:401–6. doi:10.1016/j.jclinepi.2010.07.015
