# Supplementary material for: Corticosteroid injection for plantar heel pain: a systematic review and meta-analysis
Source: BMC Musculoskelet Disord. 2019 Aug 17;20:378. doi: 10.1186/s12891-019-2749-z (PMC6698340; doi:10.1186/s12891-019-2749-z)
Supplement: Supplementary file 3 — Results of single trials that investigated pain. A summary of the findings from single trials that investigated pain but were not included in a meta-analysis. (PDF 95 kb) [file 12891_2019_2749_MOESM3_ESM.pdf]

Additional file 3. Results of single trials that investigated pain.

Comparison	Intervention		Comparator		Outcome measure	Mean difference (95% CI)*
	Mean pain (SD)	Participants	Mean pain (SD)	Participants		
**Short term pain**						
Corticosteroid injection compared to miniscalpel-needle[1]	4.0 (2.4)	30	1.6 (2.1)	31	VAS (0-10)	2.4 (1.3, 3.5)
Corticosteroid injection compared to corticosteroid injection plus foot orthoses[2]	65.3 (23.7)	22	49.3 (31.4)	28	VAS (change scores)	-16.0 (-31.3, -0.7)
Corticosteroid injection compared to a control group[3]	4.4 (2.2)	12	5.7 (2.8)	12	VAS (0-10)	-1.3 (-3.3, 0.7)
Corticosteroid injection compared to ozone injection[4]	26.0 (15.5)	15	43.3 (18.8)	15	VAS (0-10)	-17.3 (-29.6, -4.9)
Corticosteroid injection compared to prolotherapy[5]	3.2 (2.4)	40	6.9 (6.5)	40	VAS (0-10)	-3.7 (-5.8, -1.5)
**Medium term pain**						
Corticosteroid injection compared to corticosteroid injection plus physical therapy[6]	13.6 (19.1)	31	10.8 (15.1)	29	VAS)0-10)	0.2 (-0.3, 0.7)
Corticosteroid injection compared to botulinum toxin-A injection[7]	3.6 (1.9)	17	1.6 (2.1)	17	VAS (0-10)	2.0 (0.6, 3.3)
Corticosteroid injection compared to platelet-rich plasma injection[8]	81.0 (2.0)	20	95.0 (2.0)	20	AOFAS - Ankle-hindfoot Scale	14.0 (12.8, 15.2)
Corticosteroid injection compared to extracorporeal shockwave therapy[9]	84.0 (6.6)	60	85.8 (6.8)	30	MCSS	1.8 (-1.6, 5.2)
Corticosteroid injection compared to laser therapy[10]	3.8 (2.9)	30	3.2 (2.8)	24	VAS (0-10)	0.6 (-0.9, 2.1)
Corticosteroid injection compared to corticosteroid injection plus foot orthoses[2]	61.7 (28.2)	22	51.4 (28.0)	31	VAS (change scores)	-10.3 (-25.7, 5.1)
Corticosteroid injection compared to a control group[3]	3.3 (2.7)	12	6.2 (3.4)	12	VAS (0-10)	-2.9 (-5.4, -0.4)

Comparison	Intervention		Comparator		Outcome measure	Mean difference (95% CI)*
	Mean pain (SD)	Participants	Mean pain (SD)	Participants		
Corticosteroid injection compared to dry-needling[11]	0.5 (1.3)	34	1.6 (1.2)	32	VAS (0-10)	-1.0 (-1.6, -0.4)
Corticosteroid injection compared to ozone injection[4]	26.0 (19.6)	15	18.7 (11.2)	15	VAS (0-10)	7.3 (-4.1, 18.7)
Corticosteroid injection compared to prolotherapy[5]	4.4 (3.5)	40	2.8 (2.3)	40	VAS (0-10)	1.6 (0.3, 2.9)
**Longer term pain**						
Corticosteroid injection compared to corticosteroid injection plus physical therapy[6]	9.5 (15.3)	31	1.5 (2.5)	31	VAS (0-10)	0.7 (0.2, 1.2)
Corticosteroid injection compared to botulinum toxin-A injection[7]	3.8 (1.1)	17	1.1 (1.5)	17	VAS (0-10)	2.7 (1.8, 3.6)
Corticosteroid injection compared to tenoxicam injection[12]	3.1 (2.3)	30	2.9 (2.0)	31	VAS	0.2 (-0.8, 1.3)
Corticosteroid injection compared to foot orthoses[2]	63.7 (31.4)	22	50.6 (28.6)	26	VAS (change scores)	-13.1 (-30.2, 4.0)
Corticosteroid injection compared to miniscalpel-needle[1]	6.5 (2.7)	30	1.1 (1.7)	31	VAS	5.4 (4.3, 6.5)
Corticosteroid injection compared to platelet-rich plasma injection[8]	74.0 (2.3)	20	94.0 (2.2)	20	AOFAS - Ankle-hindfoot Scale	20.0 (18.6, 21.4)
Corticosteroid injection compared to laser therapy[10]	3.4 (3.0)	30	3.2 (3.3)	24	VAS (0-10)	0.2 (-1.5, 1.9)
Corticosteroid injection compared to corticosteroid injection plus foot orthoses[2]	63.7 (31.4)	22	61.3 (27.2)	28	VAS (change scores)	-2.4 (-18.9, 14.1)
Corticosteroid injection compared to prolotherapy[5]	6.8 (4.4)	40	6.5 (6.4)	40	VAS (0-10)	0.1 (-0.4, 0.5)

Abbreviations: CI, confidence interval; SD, standard deviation; VAS, visual analogue scale (0 = no pain, 10 = worst pain); AOFAS, American Orthopaedic Foot and Ankle Score (0 = worst pain, 100 = no pain); MCSS, Mayo Clinic Scoring System (0 = worst pain, 100 = no pain).

* Negative values indicate the comparison favours corticosteroid injection.

References

1. Li S, Shen T, Liang Y, Zhang Y, Bai B. Miniscalpel-needle versus steroid injection for plantar fasciitis: a randomized controlled trial with a 12-month follow-up. Evidence-Based Complement Altern Med. 2014;ID 164714. doi:10.1155/2014/164714.

2. Kriss S. Injectable steroids in the management of heel pain. A prospective randomised trial. Br J Pod. 2003;6:40–2.

3. Karimzadeh A, Raeissadat SA, Erfani Fam S, Sedighipour L, Babaei-Ghazani A. Autologous whole blood versus corticosteroid local injection in treatment of plantar fasciitis: a randomized, controlled multicenter clinical trial. Clin Rheumatol. 2017;36:661–9. doi:10.1007/s10067-016-3484-6.

4. Babaei-Ghazani A, Karimi N, Forogh B, Madani SP, Ebadi S, Fadavi HR, et al. Comparison of ultrasound-guided local ozone (O2-O3) injection vs corticosteroid injection in the treatment of chronic plantar fasciitis: a randomized clinical trial. Pain Med. 2019;20:314–22. doi:10.1093/pm/pny066.

5. Uğurlar M, Sönmez MM, Uğurlar ÖY, Adıyeke L, Yıldırım H, Eren OT. Effectiveness of four different treatment modalities in the treatment of chronic plantar fasciitis during a 36-month follow-up period: a randomized controlled trial. J Foot Ankle Surg. 2018;57:913–8. doi:10.1053/J.JFAS.2018.03.017.

6. Johannsen FE, Herzog RB, Malmgaard-Clausen NM, Hoegberget-Kalisz M, Magnusson SP, Kjaer M. Corticosteroid injection is the best treatment in plantar fasciitis if combined with controlled training. Knee Surgery, Sport Traumatol Arthrosc. 2019;27:5–12. doi:10.1007/s00167-018-5234-6.

7. Elizondo-Rodriguez J, Araujo-Lopez Y, Moreno-Gonzalez JA, Cardenas-Estrada E, Mendoza-Lemus O, Acosta-Olivo C. A comparison of botulinum toxin A and intralesional steroids for the treatment of plantar fasciitis. Foot Ankle Int. 2013;34:8–14. doi:10.1177/1071100712460215.

8. Monto RR. Platelet-rich plasma efficacy versus corticosteroid injection treatment for chronic severe plantar fasciitis. Foot Ankle Int. 2014;35:313–8. doi:10.1177/1071100713519778.

9. Saber N, Diab H, Nassar W, Razaak HA. Ultrasound guided local steroid injection versus extracorporeal shockwave therapy in the treatment of plantar fasciitis. Alexandria J Med. 2012;48:35–42. doi:10.1016/j.ajme.2011.11.005.

10. Yuzer S, Sever A, Gurcay E, Unlu E, Cakci A. Comparison of the effectiveness of laser therapy and steroid injection in epin calcanei. Turkiye Fiz Tip ve Rehabil Derg. 2006;52:68–71.

11. Rastegar S, Baradaran Mahdavi S, Hoseinzadeh B, Badiei S. Comparison of dry needling and steroid injection in the treatment of plantar fasciitis: a single-blind randomized clinical trial. Int Orthop. 2018;42:109–16. doi:10.1007/s00264-017-3681-1.

12. Guner S, Onder H, Guner SI, Ceylan MF, Gökalp MA, Keskin S. Effectiveness of local tenoxicam versus corticosteroid injection for plantar fasciitis treatment. Orthopedics. 2013;36:e1322–6. doi:10.3928/01477447-20130920-27.
