# Supplementary material for: Corticosteroid injection for plantar heel pain: a systematic review and meta-analysis
Source: BMC Musculoskelet Disord. 2019 Aug 17;20:378. doi: 10.1186/s12891-019-2749-z (PMC6698340; doi:10.1186/s12891-019-2749-z)
Supplement: Supplementary file 4 — Results of single trials that investigated ‘first step’ pain. A summary of the findings from single trials that investigated ‘first step’ pain but were not included in a meta-analysis. (PDF 109 kb) [file 12891_2019_2749_MOESM4_ESM.pdf]

Additional file 4. Results of single trials that investigated 'first step' pain.

Comparison	Intervention		Comparator		Outcome measure	Mean difference (95% CI)*
	Mean pain (SD)	Participants	Mean pain (SD)	Participants		
**Short term 'first step' pain**						
Corticosteroid injection compared to extracorporeal shockwave therapy[1]	4.6 (2.8)	20	3.4 (2.8)	20	VAS (0-10)	1.2 (-0.5, 2.9)
Corticosteroid injection compared to miniscalpel-needle[2]	4.2 (2.5)	30	1.7 (2.1)	31	VAS (0-10)	2.5 (1.3, 3.7)
Corticosteroid injection compared to platelet-rich plasma injection[3]	3.7 (1.9)	16	6.0 (2.1)	16	VAS (0-10)	-2.3 (-3.7, -0.9)
Corticosteroid injection compared to ozone injection[4]	26.7 (23.8)	15	54.7 (17.7)	15	VAS (0-10)	-28.0 (-43.0, -12.9)
Corticosteroid injection compared to foot orthoses[5]	22.6 (22.9)	50	30.6 (26.6)	53	VAS (0-10)	-9.2 (-18.8, 0.4)$
**Medium term 'first step' pain**						
Corticosteroid injection compared to platelet-rich plasma injection[3]	2.3 (2.4)	16	3.9 (1.6)	16	VAS (0-10)	-1.6 (-3.0, -0.2)
Corticosteroid injection compared to ozone injection[4]	25.3 (26.4)	15	24.7 (11.9)	15	VAS (0-10	0.6 (-14.0, 15.2)
Corticosteroid injection compared to foot orthoses[5]	35.3 (30.8)	50	19.8 (20.0)	53	VAS (0-10)	13.7 (3.9, 23.5)$
**Longer term 'first step' pain**						
Corticosteroid injection compared to miniscalpel-needle[2]	6.6 (2.4)	30	0.9 (1.3)	31	VAS (0-10)	5.7 (4.7, 6.7)
Corticosteroid injection compared to platelet-rich plasma injection[3]	5.8 (2.5)	16	1.7 (1.9)	16	VAS (0-10)	4.1 (2.5, 5.6)

Abbreviations: CI, confidence interval; SD, standard deviation; VAS, visual analogue scale (0 = no pain, 10 = worst pain).

* Negative values indicate the comparison favours corticosteroid injection.
$ An adjusted mean difference is reported.

References

1 Eslamian F, Shakouri SK, Jahanjoo F, *et al.* Extra corporeal shock wave therapy versus local corticosteroid injection in the treatment of chronic plantar fasciitis, a single blinded randomized clinical trial. *Pain Med* 2016;**17**:1722–31. doi:10.1093/pm/pnw113

2 Li S, Shen T, Liang Y, *et al.* Miniscalpel-needle versus steroid injection for plantar fasciitis: a randomized controlled trial with a 12-month follow-up. *Evidence-Based Complement Altern Med* 2014;**ID 164714**. doi:10.1155/2014/164714

3 Vahdatpour B, Kianimehr L, Moradi A, *et al.* Beneficial effects of platelet-rich plasma on improvement of pain severity and physical disability in patients with plantar fasciitis: a randomized trial. *Adv Biomed Res* 2016;**5**:179. doi:10.4103/2277-9175.192731

4 Babaei-Ghazani A, Karimi N, Forogh B, *et al.* Comparison of ultrasound-guided local ozone (O2-O3) injection vs corticosteroid injection in the treatment of chronic plantar fasciitis: a randomized clinical trial. *Pain Med* 2019;**20**:314–22. doi:10.1093/pm/pny066

5 Whittaker GA, Munteanu SE, Menz HB, *et al.* Effectiveness of foot orthoses versus corticosteroid injection for plantar heel pain: the SOOTHE randomized clinical trial. *J Orthop Sport Phys Ther* 2019;**49**:491–500. doi:10.2519/jospt.2019.8807
