# Supplementary material for: Corticosteroid injection for plantar heel pain: a systematic review and meta-analysis
Source: BMC Musculoskelet Disord. 2019 Aug 17;20:378. doi: 10.1186/s12891-019-2749-z (PMC6698340; doi:10.1186/s12891-019-2749-z)
Supplement: Supplementary file 5 — Results of single trials that investigated function. A summary of the findings from single trials that investigated function but were not included in a meta-analysis. (PDF 82 kb) [file 12891_2019_2749_MOESM5_ESM.pdf]

Additional file 5. Results of single trials that investigated function.

Comparison	Intervention		Comparator		Outcome measure	Mean difference (95% CI)*
	Mean (SD)	Participants	Mean (SD)	Participants		
**Short term function**						
Corticosteroid injection compared to placebo injection[1]	6.6 (20.3)	41	0 (20.3)	41	FHSQ function subscale	-6.6 (-15.4, 2.2)$
Corticosteroid injection compared to ozone injection[3]	76.9 (16.7)	15	73.2 (15.0)	15	FAAM activities of daily living subscale	-3.7 (-15.1, 7.7)
Corticosteroid injection compared to dry needling[4]	28.4 (11.6)	47	28.3 (8.9)	49	FFI disability subscale	0.1 (-4.0, 4.2)
**Medium term function**						
Corticosteroid injection compared to placebo injection[1]	4.1 (18.1)	41	0 (18.1)	41	FHSQ function subscale)	-4.1 (-11.9, 3.7)$
Corticosteroid injection compared to botulinum toxin-A injection[5]	84.7 (6.5)	17	94.9 (7.5)	17	FADI	10.2 (5.5, 14.9)
Corticosteroid injection compared to ozone injection[3]	78.3 (18.5)	15	90.7 (9.5)	15	FAAM activities of daily living subscale	12.4 (1.9, 22.9)
Corticosteroid injection compared to foot orthoses	83.9 (20.4)	50	87.9 (14.7)	53	FHSQ function subscale	2.3 (-3.4, 8.0)$
**Longer term function**						
Corticosteroid injection compared to physical therapy[6]	83.4 (17.3)	21	86.7 (21.9)	22	FAAM activities of daily living subscale	3.3 (-8.5, 15.1)
Corticosteroid injection compared to botulinum toxin-A injection[5]	83.0 (6.4)	17	95.0 (7.2)	17	FADI	12.0 (7.4, 16.6)
Corticosteroid injection compared to extracorporeal shockwave therapy[7]	43.2 (40.8)	40	37.0 (35.3)	39	FFI-R difficulty subscale	6.2 (-10.6, 23.0)

Comparison	Intervention		Comparator		Outcome measure	Mean difference (95% CI)*
	Mean (SD)	Participants	Mean (SD)	Participants		
Corticosteroid injection compared to dry needling[4]	43.1 (11.1)	47	28.8 (8.8)	49	FFI disability subscale	14.3 (10.3, 18.3)

Abbreviations: CI, confidence interval; SD, standard deviation; FHSQ, Foot Health Status Questionnaire (0 = best function, 100 = worst function); FAOS, Foot and Ankle Outcome Score (0 = worst function, 100 = best function); FADI, Foot and Ankle Disability Index (0 = worst function, 100 = best function); FAAM, Foot and Ankle Ability Measure (0 = worst function, 100 = best function); FFI, Foot Function Index (0 = best function, 100 = worst function); FFI-R, Foot Function Index - Revised (0 = best function, 100 = worst function).

* Negative values indicate the comparison favours corticosteroid injection.
$ An adjusted mean difference is reported.
# Raw values were reported in this trial on a 0 to 136 scale, rather than being converted to a 0 to 100 scale. To ensure consistency between trials, these values were converted to a 0 to 100 scale.

References

1. McMillan AM, Landorf KB, Gilheany MF, Bird AR, Morrow AD, Menz HB. Ultrasound guided corticosteroid injection for plantar fasciitis: randomised controlled trial. BMJ. 2012;344:e3260. doi:10.1136/bmj.e3260.

2. Yucel U, Kucuksen S, Cingoz HT, Anliacik E, Ozbek O, Salli A, et al. Full-length silicone insoles versus ultrasound-guided corticosteroid injection in the management of plantar fasciitis: a randomized clinical trial. Prosthet Orthot Int. 2013;37:471–6. doi:10.1177/0309364613478328.

3. Babaei-Ghazani A, Karimi N, Forogh B, Madani SP, Ebadi S, Fadavi HR, et al. Comparison of ultrasound-guided local ozone (O2-O3) injection vs corticosteroid injection in the treatment of chronic plantar fasciitis: a randomized clinical trial. Pain Med. 2019;20:314–22. doi:10.1093/pm/pny066.

4. Uygur E, Aktaş B, Eceviz E, Yilmazoğlu EG, Poyanli O. Preliminary report on the role of dry needling versus corticosteroid injection, an effective treatment method for plantar fasciitis: a randomized controlled trial. J Foot Ankle Surg. 2018;58:301–5. doi:10.1053/J.JFAS.2018.08.058.

5. Elizondo-Rodriguez J, Araujo-Lopez Y, Moreno-Gonzalez JA, Cardenas-Estrada E, Mendoza-Lemus O, Acosta-Olivo C. A comparison of botulinum toxin A and intralesional steroids for the treatment of plantar fasciitis. Foot Ankle Int. 2013;34:8–14. doi:10.1177/1071100712460215.

6. Celik D, Kus G, S rma SO. Joint mobilization and stretching exercise vs steroid injection in the treatment of plantar fasciitis: a randomized controlled study. Foot Ankle Int. 2016;37:150–6. doi:10.1177/1071100715607619.

7. Uğurlar M, Sönmez MM, Uğurlar ÖY, Adıyeke L, Yıldırım H, Eren OT. Effectiveness of four different treatment modalities in the treatment of chronic plantar fasciitis during a 36-month follow-up period: a randomized controlled trial. J Foot Ankle Surg. 2018;57:913–8. doi:10.1053/J.JFAS.2018.03.017.
