# Supplementary material for: Corticosteroid injection for plantar heel pain: a systematic review and meta-analysis
Source: BMC Musculoskelet Disord. 2019 Aug 17;20:378. doi: 10.1186/s12891-019-2749-z (PMC6698340; doi:10.1186/s12891-019-2749-z)
Supplement: Supplementary file 6 — Results of single trials that investigated plantar fascia thickness. A summary of the findings from single trials that investigated plantar fascia thickness but were not included in a meta-analysis. (PDF 65 kb) [file 12891_2019_2749_MOESM6_ESM.pdf]

Additional file 6. Results of single trials that investigated plantar fascia thickness.

Comparison	Intervention		Comparator		Outcome measure	Mean difference (95% CI)*
	Mean (SD)	Participants	Mean (SD)	Participants		
Corticosteroid injection compared to physical therapy[1]	3.8 (0.4)	28	4.3 (0.8)	28	Thickness (mm) using diagnostic ultrasound	-0.5 (-0.8, -0.2)
Corticosteroid injection compared to autologous blood injection[2]	3.4 (1.1)	21	4.3 (1.1)	28	Thickness (mm) using diagnostic ultrasound	-0.9 (-1.5, -0.3)
Corticosteroid injection compared to ozone injection[5]	3.8 (0.3)	15	4.6 (0.6)	15	Thickness (mm) using diagnostic ultrasound	-0.7 (-1.1, -0.4)

Abbreviations: CI, confidence interval; SD, standard deviation; mm, millimetres.

* Negative values indicate the comparison favours corticosteroid injection.

References

1 Ryan M, Hartwell J, Fraser S, *et al.* Comparison of a physiotherapy program versus dexamethasone injections for plantar fasciopathy in prolonged standing workers. *Clin J Sport Med* 2014;**24**:211–7. doi:10.1097/JSM.0000000000000021

2 Yesiltas F, Aydogan U, Parlak A, *et al.* The comparison of intralesionary steroid injection and autologous venous blood injection in patients with plantar fasciitis. *Acta Medica Mediterr* 2015;**31**:711–6.

3 Yucel U, Kucuksen S, Cingoz HT, *et al.* Full-length silicone insoles versus ultrasound-guided corticosteroid injection in the management of plantar fasciitis: a randomized clinical trial. *Prosthet Orthot Int* 2013;**37**:471–6. doi:10.1177/0309364613478328

4 Vahdatpour B, Kianimehr L, Moradi A, *et al.* Beneficial effects of platelet-rich plasma on improvement of pain severity and physical disability in patients with plantar fasciitis: a randomized trial. *Adv Biomed Res* 2016;**5**:179. doi:10.4103/2277-9175.192731

5 Babaei-Ghazani A, Karimi N, Forogh B, *et al.* Comparison of ultrasound-guided local ozone (O2-O3) injection vs corticosteroid injection in the treatment of chronic plantar fasciitis: a randomized clinical trial. *Pain Med* 2019;**20**:314–22. doi:10.1093/pm/pny066
